# A Radical Reform

**Published:** 1913-02

**Authors:** R. H. L. Bibb


### EDITORIAL DEPARTMENT.

DR. F. E. DANIEL, Editor DR. R. H. L. BIBB, Associate Editor

DEPARTMENTS { EUGENICS: Drs. M. Duggan and T. Y. Hull.

WOMAN'S DEPARTMENT: Mrs. F. E. Daniel.

### A RADICAL REFORM

### Bexar County Medical Society Remodels its By-Laws.

An unusually interesting and numerously attended meeting of the Bexar County Medical Society was held in the parlors of the St. Anthony Hotel, in San Antonio, on the night of the 16th ult., at which stringent and sweeping changes were made in the society's by-laws, by which, in the language of one of the speakers, "hereafter the sheep will be separated from the goats, and our members will be prevented from doing themselves the harm that comes from inserting their professional cards in lay journals and other publications carrying quack advertisements and announcements of abortion-producing nostrums, pile ointments, tape-worm remedies and such like."

Whether Bexar County Medical Society is the pioneer in this step in the right direction—which should be followed by every other medical organization in the land, or whether it is not, if I can properly appreciate the signs of the times—that grand old society of most excellent medical gentlemen can read the "handwriting on the wall," for the time is not very far off when the most ignorant layman, however blinded, will easily recognize the earmark of the "goats" from that which distinguishes the "sheep," and will act accordingly, as he is already doing in many parts of the country.

At some previous session of the society the matters treated of had been referred to a select committee of nine with instructions to fully investigate the subjects and to present their views in a report which should embody their recommendations in the premises. This the committee did most faithfully and most satisfactorily for, after reading, discussing, amending and adopting the recommendations by sections, the whole was adopted almost unanimously—the minor changes which the society made being somewhat more emphatic and intensive than the committee had recommended.

Hereafter it will be considered unethical—and the culpable subjected to suspension or expulsion—for a member of the Bexar County Medical Society to insert his professional card, or carry any advertisement of any sort, in any lay journal, hotel register or other public place. He may advise the public of a change of office, of an expected absence and of his return, provided such notice is not continued longer than ten days. He is prohibited doing lodge or society practice for less than the society's schedule of charges; but he may make contracts with railroads and other public utility corporations for caring for their sick and injured at special rates—this proviso to extend only to their employes, but not to their families; nor may he announce himself, publicly, as being employed by such public utility corporations.

During the discussion of the committee's report, some difference of opinion was manifested as to how best to put the recommendations into practice as well as to the necessity of reforms which it advocated, and it was amusing to hear an "old-timer", an ex-president of the society, say *sotto voce*. "I really believe if some of these fellows here had been present at the dawn of creation they would have objected to having chaos wiped out and order established instead." Be that as it may, I am quite sure that the Bexar County Medical Society is determined that its members shall be ethically righteous, and that the "Red Back" will support, with heart and hand, might and main, any reform it may inaugurate looking to the uplift of the medical profession and to the downfall of quackery. *"Ojala!"*

I regret exceedingly that several prominent members of the society did not attend the meeting. I am confident, however, that their absence was not due to a want of interest in the society's proceedings, for I know they are not made of that sort of material which would permit them to become recreant to the obligations which membership in any organization imposes.

The meetings of the society are held—by invitation of the manager—in the parlors of the St. Anthony Hotel, a courtesy to the profession which it is hoped that physicians visiting San Antonio will not forget.

R. H. L. BIBB.

